# Insights into the Toxicological Properties of a Low Molecular Weight Fraction from *Zoanthus sociatus* (Cnidaria)

**DOI:** 10.3390/md11082873

**Published:** 2013-08-13

**Authors:** Dany Domínguez-Pérez, Carlos Manlio Diaz-Garcia, Neivys García-Delgado, Yusvel Sierra-Gómez, Olga Castañeda, Agostinho Antunes

**Affiliations:** 1 CIIMAR/CIMAR, Interdisciplinary Centre of Marine and Environmental Research, University of Porto, Rua dos Bragas 289, Porto 4050-123, Portugal; E-Mail: danydguezperez@gmail.com; 2 Biology Department, Faculty of Sciences, University of Porto, Rua do Campo Alegre, Porto 4169-007, Portugal; 3 PhD Program in Biological Sciences, National Autonomous University of Mexico, Institute of Cellular Physiology, Mexico DF CP04510, Mexico; E-Mail: cmanlio@email.ifc.unam.mx; 4 PhD Program in Biomedical Sciences, National Autonomous University of Mexico, Institute of Cellular Physiology, Mexico DF CP04510, Mexico; E-Mails: neivysgd8684@gmail.com (N.G.-D.); yusvelsiego@gmail.com (Y.S.-G.); 5 Faculty of Biology, University of La Habana, 25 St 455, La Habana CP 10400, Cuba; E-Mail: castanedapasaron@gmail.com

**Keywords:** biological activity, toxins, *Zoanthus sociatus*, Anthozoa, Cnidaria, LD_50_ mice

## Abstract

The phylum Cnidaria is an ancient group of venomous animals, specialized in the production and delivery of toxins. Many species belonging to the class Anthozoa have been studied and their venoms often contain a group of peptides, less than 10 kDa, that act upon ion channels. These peptides and their targets interact with high affinity producing neurotoxic and cardiotoxic effects, and even death, depending on the dose and the administration pathway. Zoanthiniaria is an order of the Subclass Hexacorallia, class Anthozoa, and unlike sea anemone (order Actiniaria), neither its diversity of toxins nor the *in vivo* effects of the venoms has been exhaustively explored. In this study we assessed some toxicological tests on mice with a low molecular weight fraction obtained by gel filtration in Sephadex G-50 from *Zoanthus sociatus* crude extract. The gel filtration chromatogram at 280 nm revealed two major peaks, the highest absorbance corresponding to the low molecular weight fraction. The toxicological effects seem to be mostly autonomic and cardiotoxic, causing death in a dose dependent manner with a LD_50_ of 792 μg/kg. Moreover, at a dose of 600 μg/kg the active fraction accelerated the KCl-induced lethality in mice.

## 1. Introduction

The phylum Cnidaria is an ancient group of predominantly marine simple animals that comprise over 11,000 extant species [1], which share a common diagnostic feature: the cnida [2]. The cnida is a subcellular organelle-like capsular with eversible tubules [3,4] that contains the cnidocysts cells (also called cnidae). Of the three categories of cnidae (nematocysts, ptychocysts, and spirocysts), only nematocysts are found in all cnidarians [2]. This type of cnida may be associated with the production, discharge and inoculation of venoms in all cnidarians [5], whereby cnidarians are considered as the largest phylum of generally toxic animals [6].

Currently around 250 compounds from cnidarians have been identified including peptides, proteins, enzymes, protease inhibitors and non-proteinaceous substances [7]. Most cnidarians toxins have been successfully isolated from class Anthozoa, particularly from sea anemones, which is partly due by the stability of their toxins compared to jellyfish toxins [8]. To date, at least 191 proteins from sea anemones are recognized without ambiguities, considering the complete peptide sequences (or the information obtained by the translation of coding sequence submitted “CDSs” to GenBank database) and more than 80% deduced amino acid sequences for proteins over 10 kDa [9].

Most of these toxins correspond to peptides that act on voltage-gated sodium (Na_v_) and potassium channels (K_v_), whose molecular weights are between 3.5–6.5 kDa and 3–5 kDa, respectively [10]. These toxins also seem to have an universal distribution within the group, since all species tested have been found to contain toxins that are lethal or paralytic to crabs [10]. This claim is well supported by the number of toxins (62 for Na_v_ and 28 for K_v_) characterized [7]. Moreover, the new Na_v_ toxins from *Aiptasia diaphana* [11] and the two novel type 1 sea anemone K_v_ toxins from *Bunodosoma caissarum* [12] should be added to this list.

Despite sea anemones being the best studied in the phylum, the order Ceriantharia, Corallimorpharia and Zoanthiniaria, which are closely related to the order Actiniaria, have stayed unexplored regarding the presence of low molecular weight toxins. There are only few reports on *Zoanthus soociatus*, an organism belonging to the order Zoanthiniaria, which is known to present organic compounds such as palytoxin [13] and some alkaloids affecting platelet aggregation [14]. Other biological properties have been described for *Z. sociatus* preparations, for example, an antifilarial activity by a chloroform-methanol extract [15] and an inhibitory effect on Ca^2+^ influx in rat β-cells by a low molecular weight fraction [16]. The latter suggest that there are unraveled biological activities in *Z. sociatus* that could account for its toxicity *in vivo*. In the present study we obtained a low molecular weight fraction from *Z. sociatus* and assessed its toxicological properties in OF-1 mice. A dose-mortality curve was constructed and conspicuous toxic symptoms were monitored and discussed.

## 2. Results and Discussion

**Figure 1:**
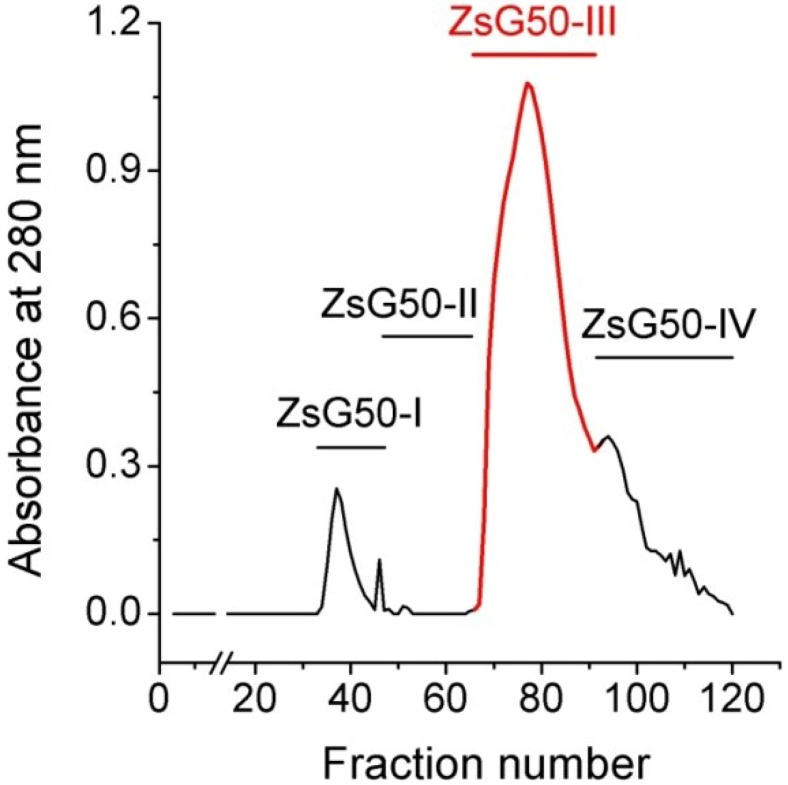
Sephadex G50 gel filtration chromatogram of *Z. sociatus* crude extract. Fractions in the chromatogram at 280 nm, obtained by Sephadex G50 gel filtration, were pooled in four major fractions. The fraction comprising the most prominent peak was named ZsG50-III and used for further toxicological studies.

*Z. sociatus* crude extract was subjected to a Sephadex G50 gel chromatography and the elution of its components was monitored by absorbance at 280 nm. The chromatogram exhibited various peaks that were pooled in four major fractions (Figure 1). The fraction ZsG50-III contained the most prominent peak and was selected for further studies because it has been demonstrated to contained low molecular weight toxins acting on ion channels in other studies on sea anemones using a similar methodology [17].

The presence of signals detected by matrix assisted laser desorption/ionization time-of-fly/time-of-fly MALDI-TOF/TOF mass spectra (MS) corresponding to *m*/*z* ratio from 700 to 6000 Da. The highest relative abundance corresponds to compounds below 1000 Da (Supplemental Figure S1) that do not seem to be peptides (Supplemental Figures S2 and S3). However, some minor peaks between 2000 and 4000 Da captured and analyzed by MS/MS analysis in reflector positive mode showed typical fragmentation of peptides (Supplemental Figure S4). Molecular weights of these peptides are in the range reported for various toxins; however blast analysis showed no significant similarity with any toxins from the UniProt database. Further procedures in the isolation of pure peaks is required in order to eliminate possible interferences in the detection of peptide signals by the main metabolites in the fraction.

To estimate the range of toxicity of the ZsG50-III fraction was started at a dose (150 μg/kg) and monitored the symptoms increasing in three-fold steps, until lethality was observed. Mice inoculated intraperitoneally with 150 μg/kg of the fraction of interest showed the same typical grooming activity of controls after injection. In the 450 μg/kg dose, animals decrease their exploratory activity 10 min after injection and remained near the walls of the cage. This symptom was accompanied with disordered breathing. At 1350 μg/kg these toxic effects were observed in less than a minute after inoculation, causing spasms, palpitations, convulsions and dead after 2 min. These effects were observed before sudden dead, which was preceded by dyspnea and reduced motile activity, suggesting cardiac arrest as the main cause of lethality. Certain drugs have been reported to cause respiratory and cardiovascular complications before cardiac arrest in mammals [18], including some cnidarian toxins [19]. It may be possible that the lethal effects were caused mainly by non-peptide toxins abundant in the fraction with molecular weight below 1000 Da. However the presence of peptide toxins that can be acting synergically should not be discarded.

**Figure 2:**
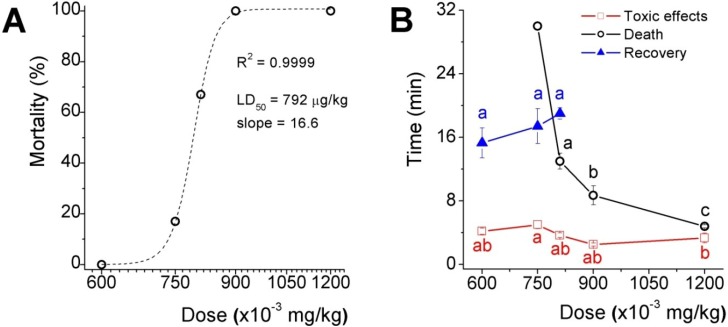
Acute toxicity assay of the low molecular weight fraction (ZsG50-III) from *Z. sociatus* crude extract. (**A**) Mortality *versus* dose curve. The plot shows a sigmoid equation fitting with the LD_50_ and the slope factor for the low molecular weight fraction from *Z. sociatus*; (**B**) The plot shows the time to listed events at each dose. The appearance of cardiovascular symptoms was common to all doses, and around 5 min after inoculation. At the lower dose, there was a time window of 10 min approximately between the onset of these effects and their disappearance. Symbols represent mean ± SEM. Letters represent statistical differences for a *p* < 0.05.

The acute toxicity results of five doses selected in the range between the two higher doses assayed in the preliminary test is shown as the percentage of lethality *versus* the dose plot fitted to a dose-response sigmoid curve (Figure 2A), which LD_50_ was 792 μg/kg and the slope factor was 16.6 (expressing the dose as mg/kg). Toxicological effects appeared in less than 5 min after inoculation in mice from all groups, recovery, however, delayed proportionally to dose in those groups where lethality were not absolute and the time to death decreased in an exponential fashion (Figure 2B). It is worth to mention that 33 and 50% of mice inoculated with the two higher doses, presented fecal and urinary incontinency, perhaps because of relaxation of sphincter smooth muscle. 

Considering that an impairment of Ca^2+^ fluxes has been previously reported for low molecular weight compounds in *Z. sociatus* crude extract in excitable cells [16] and that voltage dependent Ca^2+^ channels are relevant to cardiac function [20], we decided to explore the effect of ZsG50-III fraction on KCl-induced cardiac arrest in mice, as it is known that hyperkalemia can produce cardiac arrest [21]. 

**Figure 3:**
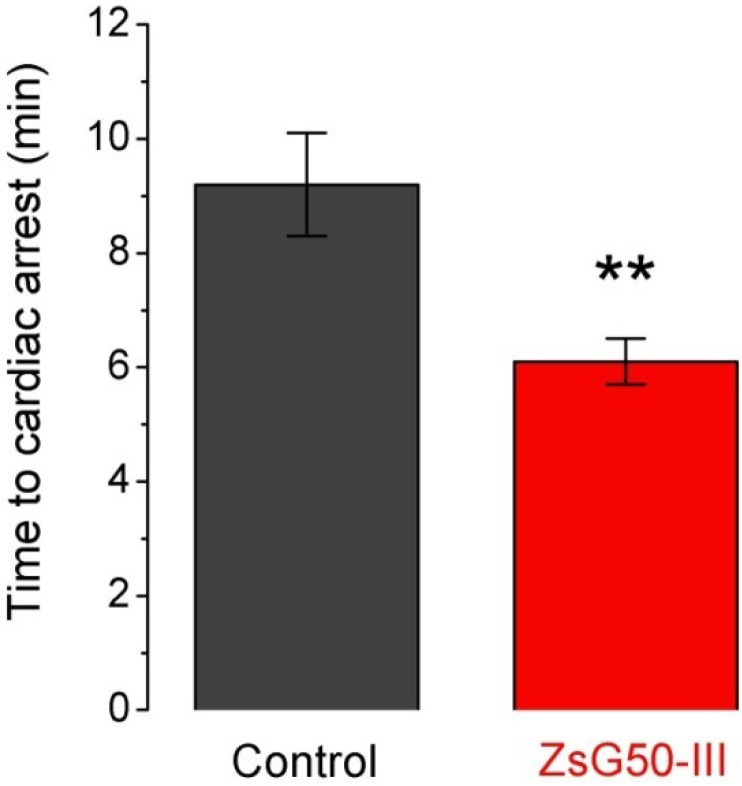
The low molecular weight fraction ZsG50-III accelerated the KCl-induced time to cardiac arrest. Bars represent the time to cardiac arrest after inoculation of a lethal dose of KCl (1000 mg/kg) in controls (*N* = 6) and simultaneous administration of KCl and 600 μg/kg of the low molecular weight fraction in the ZsG50-III treated group (*N* = 7). Bars represent mean ± SEM. ** *p* < 0.01.

Seven mice were inoculated with KCl (1000 mg/kg) and six of them (86%) presented sudden death after 9.2 min in average (Figure 3). A similar dose of NaCl was applied to another group of six mice and no lethality was observed suggesting that cardiac arrest was mediated by high K^+^ instead an hyperosmotic load. To analyze if the fraction ZsG50-III could modify the lethal effect of KCl, we used a non-lethal dose of the fraction (600 μg/kg) and compared the times to cardiac arrest respect to the KCl only group. All mice treated with KCl and ZsG50-III died in an average time of 6.1 min, which indicated that the fraction of interest accelerated KCl-cardiac arrest. Moreover, the controls with NaCl in this condition (*N* = 6) did not presented mortality and the symptoms were similar to the observed in the acute toxicity test for the same dose of the fraction in physiological saline.

The acceleration of KCl-induced cardiac arrest by ZsG50-III suggests that the fraction could enhance the KCl-mediated cardiac dysfunction. It has been reported that *Z. sociatus* crude extract contains low molecular weight compounds that inhibit Ca^2+^ influx to pancreatic beta cells and impairs glucose tolerance in rats [16]. Interestingly it is well known that overdose of Ca^2+^ channel blockers cause cardiovascular failure [22,23] but also hyperglycemia as a result, in part, of insulin secretion impairment [24]. In the light of previous studies and our results, a malfunction of Ca^2+^ fluxes could account for the observed cardiac toxicity during administration of high doses of ZsG50-III. However, further efforts should be made to unravel the exact mechanisms and to identify the responsible molecules.

## 3. Experimental Section

### 3.1. General Procedures

*Z. sociatus* were provided by the National Aquarium of Cuba, La Habana, Cuba. The zooids were brought to the Laboratory alive and kept in clean seawater until crude extract preparation. Sample collection and crude extract preparation. Briefly, specimens were cut in small pieces after removal of their stolonal bases and blended in distilled water at 4 °C. The whole-bodies homogenate was filtered in a spun glass mesh to remove large pieces of tissue and the filtrate was centrifuged twice in a Beckmann CS-6RK centrifuge at 1376× *g* during 30 min at 4 °C. Finally, the supernatants were recovered and freeze-dried. 

### 3.2. Gel Filtration

The low molecular weight fraction from *Z. sociatus* was obtained by gel filtration chromatography of crude extract in Sephadex G50 matrix (Amersham Pharmacia Biotech, Uppsala, Sweden). Two grams (2 g) of crude extract were dissolved in 20 mL of 0.1 M ammonium acetate buffer (pH 6.7), centrifuged as described above and the supernatant was filtered through a 0.22 μm membrane (Merck Millipore, Billerica, MA, USA). The filtrate was applied to a chromatographic column (3.3 × 84 cm), packed with a Sephadex G50 matrix and previously equilibrated with the same ammonium acetate buffer. Chromatography was performed at a constant linear flow rate of 3.9 cm/h collecting fractions of 8.4 mL while monitoring elution through absorbance at 280 nm. Collected fractions were pooled in four major fractions and the third, usually the most prominent and containing low molecular weight compounds, was freeze-dried and used for experiments. The protein concentration was assessed using a bicinchoninic acid kit (Thermo Scientific, Rockford, IL, USA).

### 3.3. Mass Spectrometry Analysis

To evaluate the complexity of the fraction of interest MALDI-TOF/TOF (4800 Plus MALDI-TOF/TOF Analyzer; AB SCIEX, Framingham, MA, USA) spectra were obtained in positive linear mode from 700 Da to 12,000 Da and reflector positive mode 700–4000 Da using the matrix α-cyano-4-hydroxycinnamic acid (α-CHCA) and also sequencing of peptide/metabolite in MS/MS mode. Samples were previously concentrated and cleaned according to the manufacturer’s instructions on a micro C18 ZipTiP column (Millipore, Bedford, MA, USA). Afterwards, some of these peptides were selected to cleave for partial sequencing.

### 3.4. Acute Toxicity Test

Five groups of six OF-1 male mice were intraperitoneally (i.p.) administered with three doses of the fraction of interest: 150, 450 and 1350 μg/kg in three 18–22 g OF-1 male mice each. Previous to the inoculation the fraction was dissolved in physiological saline (0.9% NaCl solution) and controls received a similar volume of vehicle alone. The percentage of mortality as a function of the dose was fitted to a Dose-Response curve to determine the mean Lethal Dose (LD_50_) and the slope factor. Observations were done during the first hour post inoculation and the control and the surviving mice were monitored again after 24 h. Controls received a similar volume of saline and no dead was recorded. OF-1 mice were obtained from National Center for the Production of Laboratory Animals (CENPALAB), Cuba. Animal manipulation was performed according to the “International Guiding Principles for Biomedical Research Involving Animals” (Council for International Organizations of Medical Sciences, Geneva, Switzerland, 2012). 

### 3.5. Data Analysis

Comparisons were performed by the non-parametric Mann-Whitney and Kruskal-Wallis (with a post-hoc Dunn) tests using GraphPad InStat version 3.00 (GraphPad Software, San Diego, CA, USA). Graphics were constructed using Origin version 7 (OriginLab Corporation, Northampton, MA, USA), as well as dose-response curve fitting.

## 4. Conclusions

The present study shows novel toxicological effects *in vivo* of a low molecular weight fraction from *Zoanthus sociatus* crude extract. MALDI-TOF mass spectra confirm that the low molecular weight fraction is composed by a mixture of non-peptides and peptides compounds between 700 and 6000 Da that caused signs of toxicity mainly related with cardiorrespiratory impairment and autonomic symptoms. The percentage of lethality showed a dose-dependent relation. Interestingly, our fraction of interest accelerated KCl-cardiac arrest, suggesting that the fraction could enhance the KCl-mediated cardiac dysfunction. Our report adds insights to the few studies related with this species and the potentialities of its low molecular weight compounds. However, further studies on this fraction should be performed into the composition of this fraction with the aim of elucidating the fraction components and possible mechanisms of action that support its toxicological properties.

## Supplementary Files

Supplementary File 1 Supplemental Information (PDF, 240 KB)
