# Supplementary material for: Insights into the Toxicological Properties of a Low Molecular Weight Fraction from Zoanthus sociatus (Cnidaria)
Source: Mar Drugs. 2013 Aug 13;11(8):2873–81. doi: 10.3390/md11082873 (PMC3766870; doi:10.3390/md11082873)

Supplemental Information

Figure S1. Matrix assisted laser desorption/ionization time-of fly/time-of-fly (MALDI-TOF/TOF) mass spectra (MS) of the fraction ZsG50-III obtained in positive linear mode from 700 Da to 12,000 Da. The majority of the signal corresponded to compounds with molecular weight below 1000 Da.

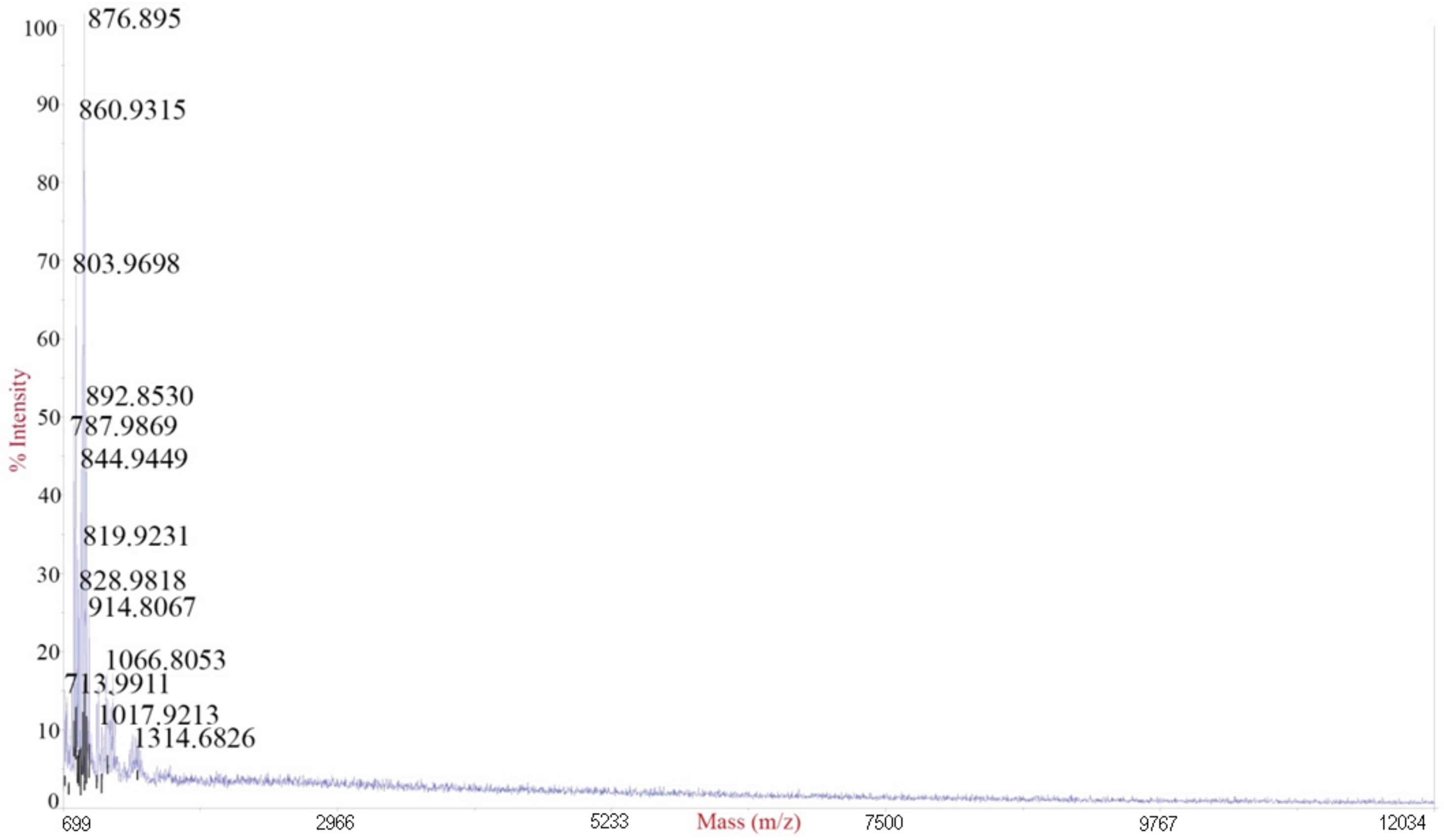

Figure S2. Sequencing profile of peak 876.98 of MALDI-TOF/TOF MS of the fraction ZsG50-III.

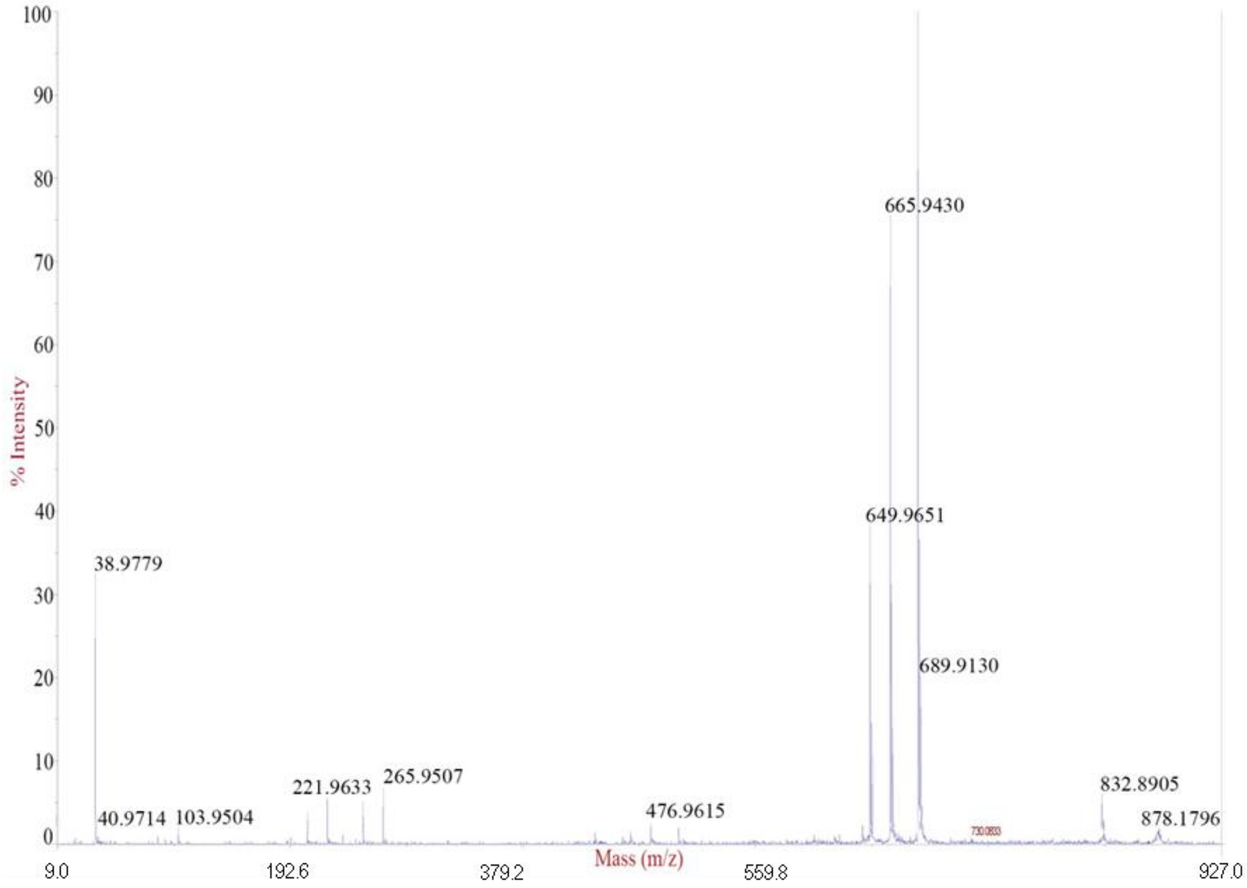

Figure S3. Sequencing profile of peak 1066.8053 of MALDI-TOF/TOFMS of the fraction ZsG50-III.

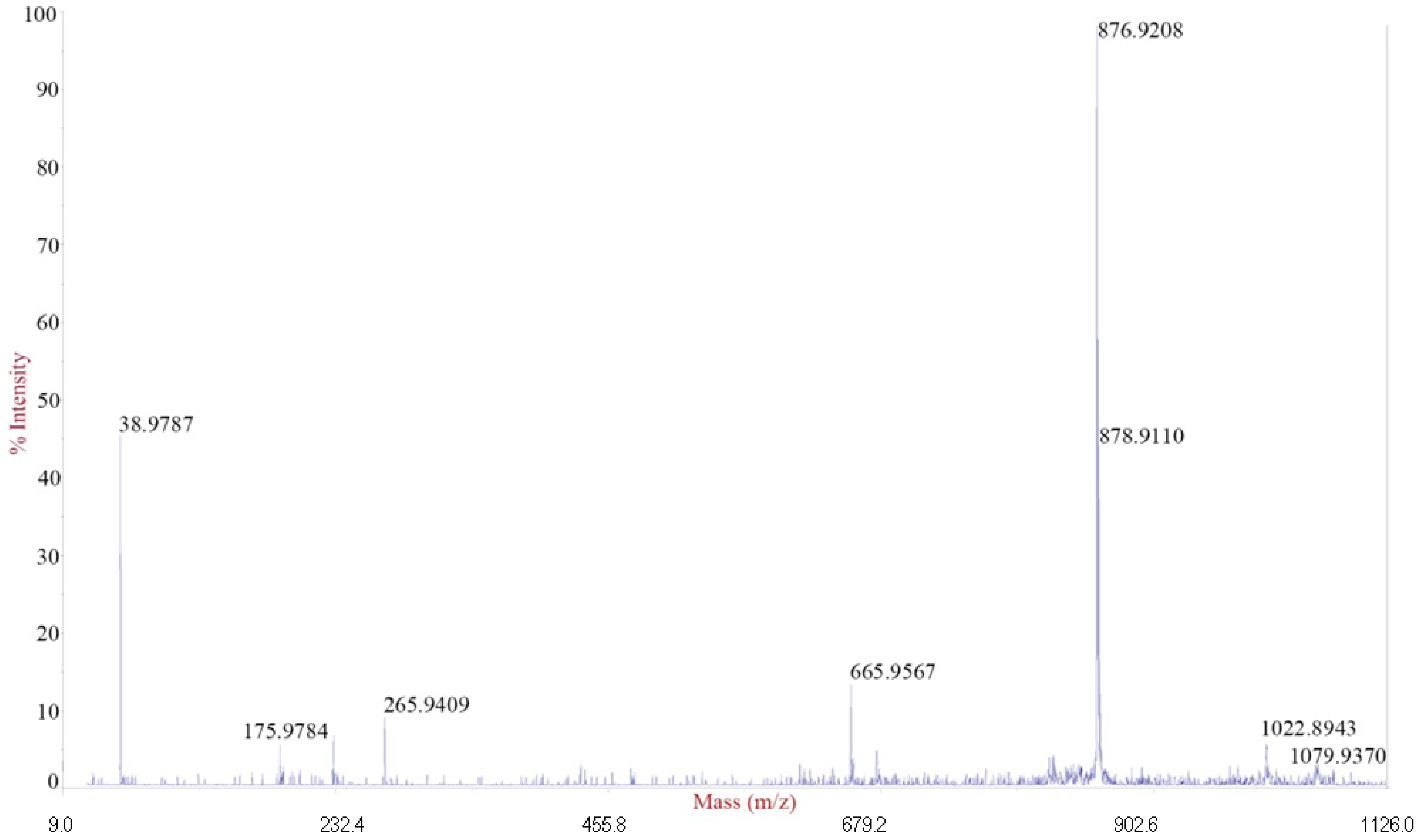

Figure S4. MS/MS analysis in reflector positive mode of minor peaks between 2000 and 4000 Da, obtained by MALDI-TOF/TOF MSfrom the fraction ZsG50-III.

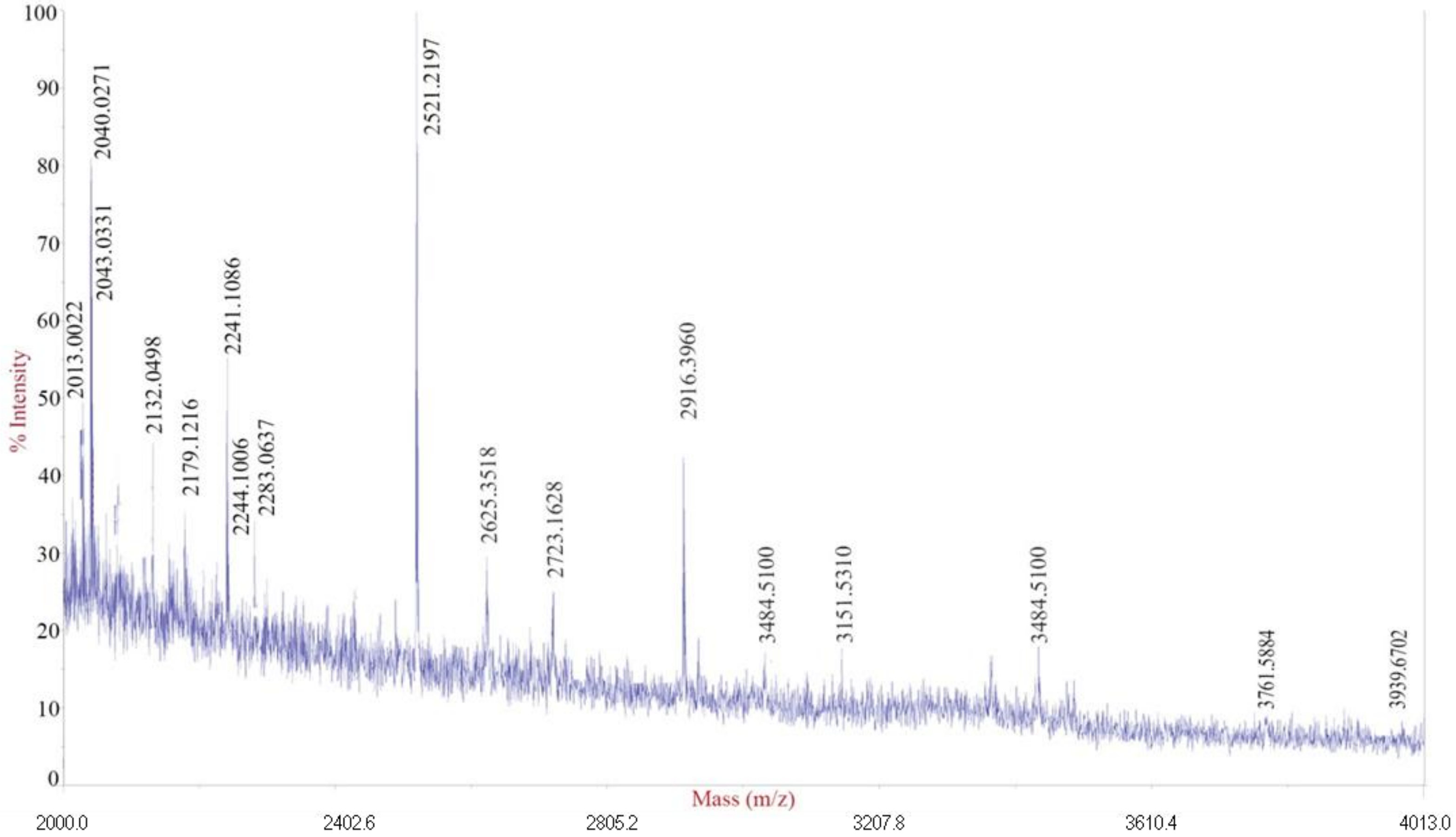

Supplement: Supplementary File 1 — Supplemental Information (PDF, 240 KB) [file marinedrugs-11-02873-s001.pdf]
